# Host-Induced Silencing of *Fusarium graminearum* Genes Enhances the Resistance of *Brachypodium distachyon* to *Fusarium* Head Blight

**DOI:** 10.3389/fpls.2019.01362

**Published:** 2019-10-30

**Authors:** Fuxin He, Ruiming Zhang, Jiaxin Zhao, Tuo Qi, Zhensheng Kang, Jun Guo

**Affiliations:** State Key Laboratory of Crop Stress Biology for Arid Areas and College of Plant Protection, Northwest A&F University, Yangling, China

**Keywords:** *Brachypodium distachyon*, *Fusarium graminearums*, CYP51, essential protein kinase, host-induced gene silencing (HIGS)

## Abstract

*Fusarium* head blight (FHB) caused by *Fusarium* pathogens are devastating diseases worldwide. Host-induced gene silencing (HIGS) which involves host expression of double-stranded RNA (dsRNA)-generating constructs directed against genes in the pathogen has been a potential strategy for the ecological sound control of FHB. In this study, we constructed transgenic *Brachypodium distachyon* lines carrying RNA interference (RNAi) cassettes to target two essential protein kinase genes *Fg00677* and *Fg08731*, and cytochrome P450 lanosterol C14-α-demethylase (CYP51) encoding genes (*CYP51A*, *CYP51B*, and *CYP51C*) of *Fusarium graminearum*, respectively. Northern blotting confirmed the presence of short interfering RNAs (siRNA) derived from *Fg00677*, *Fg08731*, and *CYP51* in transgenic *B. distachyon* plants, and the transcript levels of the corresponding genes were down-regulated in the *F. graminearum* colonizing *B. distachyon* spikes. All the corresponding independent, *Fg00677*-RNAi, *Fg08731*-RNAi, and *CYP51*-RNAi transgenic T2 lines exhibited strong resistance to *F. graminearum*, suggesting that silencing molecules produced by transgenic plants inhibited the corresponding gene function by down-regulating its expression, thereby reducing pathogenicity. Our results indicate that *Fg00677* and *Fg08731* are effective targets for HIGS and can be applied to construct transgenic HIGS materials to enhance FHB resistance in wheat and other cereal crops.

## Introduction

*Fusarium* head blight (FHB), which is caused by the fungal pathogen *Fusarium graminearum*, is a devastating disease in wheat production around the world (Osborne and Stein, 2007). Wheat can be infected by *F. graminearum* from the seedling to the heading stage, causing seedling blight, head blight, basal stem rot, and stalk rot (Stenglein, 2009). *F. graminearum* produces mycotoxins, such as deoxynivalenol, that accumulate in wheat grains, detrimentally affect the food quality and thus pose a serious threat to human and animal health (Zhang et al., 2013). Current control methods are mainly dependent on chemical control, but fungicide resistance has become increasingly prominent due to long-term use of a single chemical fungicide (Chen et al., 2007). Breeding new wheat cultivars for resistance against FHB is the most effective measure to control the disease. Due to the narrow genetic base of wheat, breeding new cultivars against FHB by traditional breeding methods has been difficult (Liu et al., 2009; Loffler et al., 2009). Therefore, a more effective and stable method for enhancing the resistance against FHB must be developed.

RNA interference (RNAi) is extensively used in functional genomics studies (Carthew, 2001; Ketting, 2011). The RNAi process includes three steps. First, a double-stranded RNA (dsRNA) or a single-stranded RNA that can form a hairpin RNA (hpRNA) is recognized by the ribonuclease Dicer and cleaved into small interfering RNAs (siRNAs) (Fagard et al., 2000). Then, siRNAs are processed into the RNA-induced silencing complex (RISC) containing an Agonaute protein (Fagard et al., 2000). Finally, the RISC binds to the target mRNA through homologous pairing, down-regulating gene expression at the transcriptional or post-transcriptional level (Ghildiyal and Zamore, 2009; Liu and Paroo, 2010). The expression of hpRNA, dsRNA, or siRNA molecules directed against parasite transcripts has been used to decrease these parasite transcripts and then to control harmful parasitic organism in plants (Cai et al., 2018); this is referred to as host-induced gene silencing (HIGS) (Nunes and Dean, 2012). Later studies have confirmed that HIGS can be a new, pesticide-free, and potentially sustainable option to enhance resistance of crops against bacteria, viruses, fungi, insects, nematodes, and parasitic weeds (Patil et al., 2011; Saurabh et al., 2014; Zhu et al., 2017; Cai et al., 2018; Panwar et al., 2018; Qi et al., 2018). HIGS has been an effective strategy to confer resistance against *Fusarium* pathogens *F. graminearum*, *F. oxysporum* and *F. culmorum* (Koch et al., 2013; Ghag et al., 2014; Cheng et al., 2015; Hu et al., 2015; Chen et al., 2016). However, there are only a few studies on the application of HIGS to enhance the resistance against FHB in wheat, and it is urgent to identify new effective HIGS target genes in *F. graminearum*.

Due to the special challenges of wheat genetic transformation, including a long process, cumbersome procedures, and high cost, it will be beneficial to use effective HIGS targets for genetic transformation. Quickly determining the most effective HIGS target is the primary problem for developing wheat disease-resistant materials. As a monocotyledon that is widely grown in the temperate zone, *Brachypodium distachyon* is rich in germplasm resources. *B. distachyon* has become an ideal model plant for functional genomics study of monocotyledons because of its interesting characteristics, including a small size, a short life-cycle, a small genome, diploid inheritance, self-fertility, a routine genetic transformation procedure, and simple growth requirements (Mur et al., 2011). *B. distachyon* is closely related to the large-genome cereal grasses, such as rice and wheat, and it is more closely related evolutionarily to wheat than to rice (Draper et al., 2001). *B. distachyon* is the ideal model plant to study the mechanism of plant-pathogen interactions because it is a host for many cereal pathogens (Peraldi et al., 2011; Sandoya and de Oliveira Buanafina, 2014). For example, *B. distachyon*-*F. graminearum* interactions closely model the head blight in wheat caused by *F. graminearum* (Peraldi et al., 2011). Therefore, we tested whether *B. distachyon* can be used for rapidly identifying effective HIGS targets that will be applied in developing FHB resistance in wheat.

In our current study, we selected two genes (*Fg00677* and *Fg08731*) as targets for HIGS analysis. *Fg00677* and *Fg08731*, which encode alpha catalytic subunit of casein kinase 2 (CK2) and casein kinase 1 (CK1), respectively, are essential in *F. graminearum* and are up-regulated when *F. graminearum* infects wheat spikes (Wang et al., 2011). As the resistance to *F. graminearum* was enhanced through HIGS methods targeting *CYP51* genes in barley and *Arabidopsis* (Koch et al., 2013), *CYP51* was selected as a HIGS target. We show that the transgenic *B. distachyon* expressing *Fg00677*-RNAi, *Fg08731*-RNAi, or *CYP51*-RNAi construct confers resistance to *F. graminearum*, indicating that *Fg00677* and *Fg08731* can be used as ideal targets to enhance the resistance to FHB in wheat by HIGS methods, and HIGS applied in the *B. distachyon*-*F. graminearum* interaction is a valuable model to rapidly identify effective HIGS targets.

## Materials and Methods

### Plant Growth Conditions and Manipulations

*B. distachyon* Bd21-3 was cultivated in a growth chamber at 22°C with a 16 h light/8 h darkness photoperiod. *F. graminearum* strain PH-1 was cultured on Potato Dextrose Agar Medium (PDA). Conidia were produced in Carboxymethyl Cellulose (CMC) broth as described previously (Duvick et al., 1992).

### Sequence Alignments and Phylogenetic Analysis

The sequence information of genes studied in this paper was acquired from Ensembl Fungi (http://fungi.ensembl.org/Fusarium_graminearum_gca_000240135/Info/Index). Gene numbers of *F. graminearum* genes were abbreviated by replacing FGSG_ with Fg. The sequences of Fg00677 and Fg08731 were analyzed using BLAST search, then the conserved domain of Fg00677 and Fg08731 was detected with InterProScan and NCBI Conserved Domain Search. Multiple sequence alignment was implemented with DNAMAN and CLUSTALX2.0 software. The phylogenetic tree was constructed with the MEGA 5.0 software (Tamura et al., 2011).

### RNAi Vector Construction

Specific partial sequences of *CYP51A* (*Fg04092*), *CYP51B* (*Fg01000*), and *CYP51C* (*Fg11024*) were used to prepare the *CYP51*-RNAi constructs as described by Koch et al. (2013). The 294 bp of *CYP51A*, 220 bp of *CYP51B*, and 238 bp of *CYP51C* were stacked into CYP51BAC by overlap PCR using the predesigned primer (**Figure 2A**; **Table S1**). The sequence-specific fragments of *Fg00677* and *Fg08731* used for the preparing the RNAi constructs were amplified from *F. graminearum* complementary DNA (cDNA) by PCR using the primers shown in **Table S1**. The length of *Fg00677*, *Fg08731*, and *CYP51BAC* fragments were 452, 481, and 752 bp, respectively (**Figure 2B**; **Figure S2**). These sequence-specific fragments were cloned into the gateway^™^ pDONR221 vector to generate the entry constructs (pDONR221-*Fg00677*, pDONR221-*Fg08731*, and pDONR221-*CYP51BAC*) by gateway BP reactions. The recombinant entry module can be used in gateway LR reaction to transfer the sequence-specific fragment to the pCAMBIA1300-based vector to generate *Fg00677*-RNAi, *Fg08731*-RNAi, and *CYP51*-RNAi construct, respectively **Figure 2B**.

### Genetic Transformation of *B. distachyon*

The RNAi vectors were transformed into *Agrobacterium tumefaciens* strain AGL1 through the electroporation method (Lazo et al., 1991). The *A. tumefaciens* was cultured in mannitol glutamate/luria-bertani (MG/L) medium (5 g/L tryptone, 2.5 g/L yeast extract, 5 g/L NaCl, 5 g/L mannitol, 1.2 g/L glutamic acid, 0.25 g/L K_2_HPO_4_, 0.1 g/L MgSO_4_, pH to 7.2 with 1N NaOH) supplemented with 50 mg/L rifampicin and 50 mg/L kanamycin to an OD600 of 0.6. The constructs contained a hygromycin phosphotransferase gene to confer hygromycin resistance to transformed plants. The Bd21-3 wild-type line was genetically transformed using the procedure of Vogel and Hill (2008).

### Pathogenicity Assays

Point inoculation and evaluation of symptoms followed the procedure of Pasquet et al. (2016) and Peraldi et al. (2011). Point inoculation was performed by pipetting 300 conidia in 0.01% Tween-20 (3 µL of a 10^5^ conidia per mL suspension) into a central floral cavity of the second spikelet, numbered from the top of the spike of different lines in mid-anthesis stages (approximately 40–45 days after sowing). Inoculated plants were covered with clear plastic bags. During the first 24 h, inoculated heads were kept in the dark, then incubated with a photoperiod of 16 h light/8 h darkness at 25°C with the same light intensities as those used for plant development. Application of 0.01% Tween-20 was performed as the control condition for each inoculation experiment. At 9 days after inoculation, symptoms were evaluated with a scoring scale for each inoculated spike from 0 to 4 as follows: 0, no symptoms; 1, only the inoculated floret was symptomatic (most frequently, only browning); 2, extension of symptoms to additional florets of the inoculated spikelet (browning or bleaching); 3, symptoms on the entire inoculated spikelet; and 4, extension of the symptoms to the entire inoculated spikelet and at least one adjacent spikelet. Three independent biological replicates were performed.

### DNA and RNA Isolation, PCR, qRT-PCR, and Northern Blotting

Plant DNA was isolated by the cetyltrimethylammonium bromide (CTAB) method (Hormaza, 2002). The transgenic plants were identified by PCR using gene-specific primers (00677-RNAi-F/00677-RNAi-R, 08731-RNAi-F/08731-RNAi-R, and CYPBAC-RNAi-F/CYPBAC-RNAi-R) and universal primer (Bar-F/Bar-R) (**Table S1**). Total RNA was extracted from spikelets using Trizol reagent (Invitrogen, Carlsbad, CA, USA) according to the manufacturer’s instructions and transcribed into cDNA for quantitative real-time PCR (qRT-PCR). qRT-PCR analysis was carried out with a CFX96TM Real-Time PCR machine (Bio-Rad). Transcript levels of *Fg00677*, *Fg08731*, *CYP51A*, *CYP51B*, and *CYP51C* were measured using cDNA prepared from total RNA isolated from five spikes of every corresponding transgenic line or non-transformed control plant inoculated with *F. graminearum* by qRT-PCR as described previously (Pasquet et al., 2016). Specific primers for qRT-PCR analysis of each gene were designed using the Primer 5.0 program (**Table S1**). Quantification results were analyzed using the comparative 2^-ΔΔCT^ method. To assess fungal transcript levels, the *F. graminearum beta*-*tubulin* gene was used as the normalizing reference gene. Three independent biological replicates were performed. To quantify fungal biomass, the ratio of single copy *beta*-*tubulin* and the *BdUBC18* was assessed in genomic DNA isolated from infected spikes using gene-specific primers (**Table S1**) (Cheng et al., 2015; Wang et al., 2017). The relative amounts of PCR product of *beta*-*tubulin* and *BdUBC18* in *F. graminearum*-infected samples were calculated using generated gene-specific standard curves to quantify the *F. graminearum* and *B. distachyon* gDNA, respectively. Three independent biological replicates were performed. Northern blotting was used to detect accumulation of siRNAs as described previously (Zhu et al., 2017). The fragments derived from *Fg00677*, *Fg08731*, and *CYP51BAC* used in RNAi vector construction were produced by PCR, and then used as probe labeled by the random priming method to detect the siRNA derived from the transgenic RNAi plants (**Supplementary Table S1**). The isolated total RNAs were separated in 19% polyacrylamide/7M urea gels and transferred to Hybond N^+^ membranes (Amersham) using a mini trans-blot (Bio-Rad, Hercules, CA). The membranes were UV cross-linked in a UV crosslinker (CX-2000, UVP, Upland, CA, USA). The membranes were prehybridized with PerfectHyb TM (Sigma-Aldrich, St. Louis, MO, USA), and hybridized with the P^32^-labeled DNA probes overnight in PerfectHyb buffer.

## Results

### Sequence Analysis of *Fg00677* and *Fg08731*

**Figure 1:**
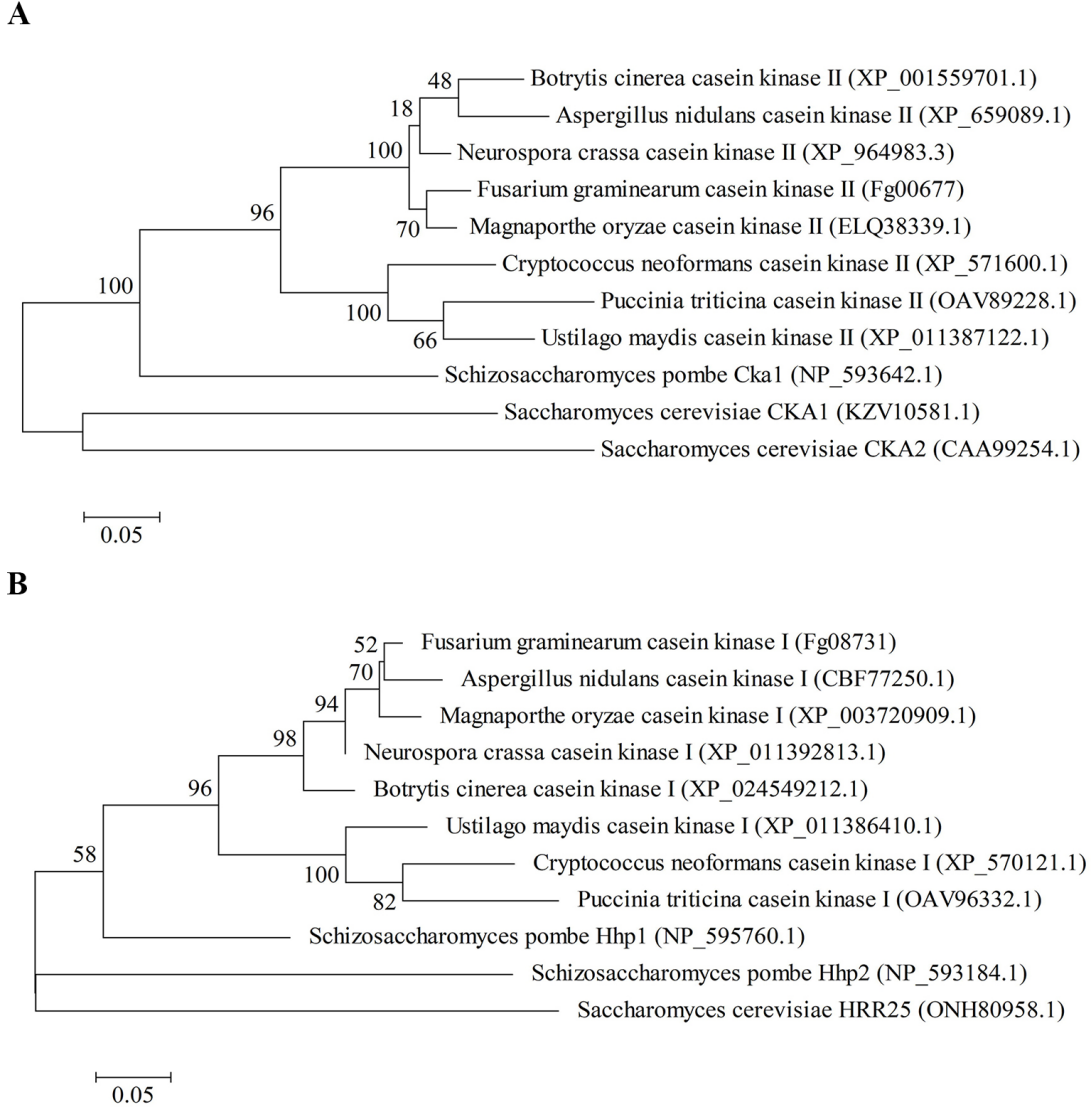
Phylogenetic analysis of Fg00677 **(A)** and Fg08731 **(B)** with their homologs in other fungal species. Phylogenetic analysis was carried out with the MEGA5 software by the maximum likelihood tree-building methods.

*Fg00677* and *Fg08731* encode the alpha catalytic subunit of CK2 and CK1, respectively (Wang et al., 2011). Sequence analysis indicated that *Fg00677* has an open reading frame (ORF) of 1,023 bp, encoding a putative protein composed of 340 amino acids with a molecular weight of 39.72 kDa and an isoeletric point (pI) of 7.55. *Fg08731* has an ORF of 1,194 bp, encoding a putative protein composed of 397 amino acids with a molecular weight of 45.05 kDa and a pI of 9.58. The multi-sequence alignment of CK2 proteins from different organisms in NCBI database revealed that Fg00677 is 85%, 73%, 50%, 55%, and 64% identical to *Aspergillus nidulans* CK2, *Ustilago maydis* CK2, *S. cerevisiae* CKA1, *S. cerevisiae* CKA2, and *Schizosaccharomyces pombe* Cka1, respectively, and contain the STKc_CK2_alpha conserved domain (**Figure S1A**). The multi-sequence alignment of CK1 proteins from different organisms in NCBI database revealed that Fg08731 is 82%, 67%, 64%, 52%, and 43% identical to *A. nidulans* CK1, *U. maydis* CK1, *S. pombe* Hhp1, *S. pombe* Hhp2, and *S. cerevisiae* HRR25, respectively, and contain the STKc_CK1_delta_epsilon conserved domain (**Figure S1B**). Phylogenetic analysis confirmed that Fg00677 is orthologous to *A. nidulans* CK2, *U. maydis* CK2, *S. cerevisiae* CKA1, *S. cerevisiae* CKA2, and *S. pombe* Cka1 (**Figure 1A**), whereas Fg008731 is orthologous to *A. nidulans* CK1, *U. maydis* CK1, *S. pombe* Hhp1, *S. pombe* Hhp2, and *S. cerevisiae* HRR25 (**Figure 1B**). These results indicate that Fg00677 and Fg08731 are highly conserved in filamentous fungi.

### Genetic Transformation of *B. distachyon*

**Figure 2:**
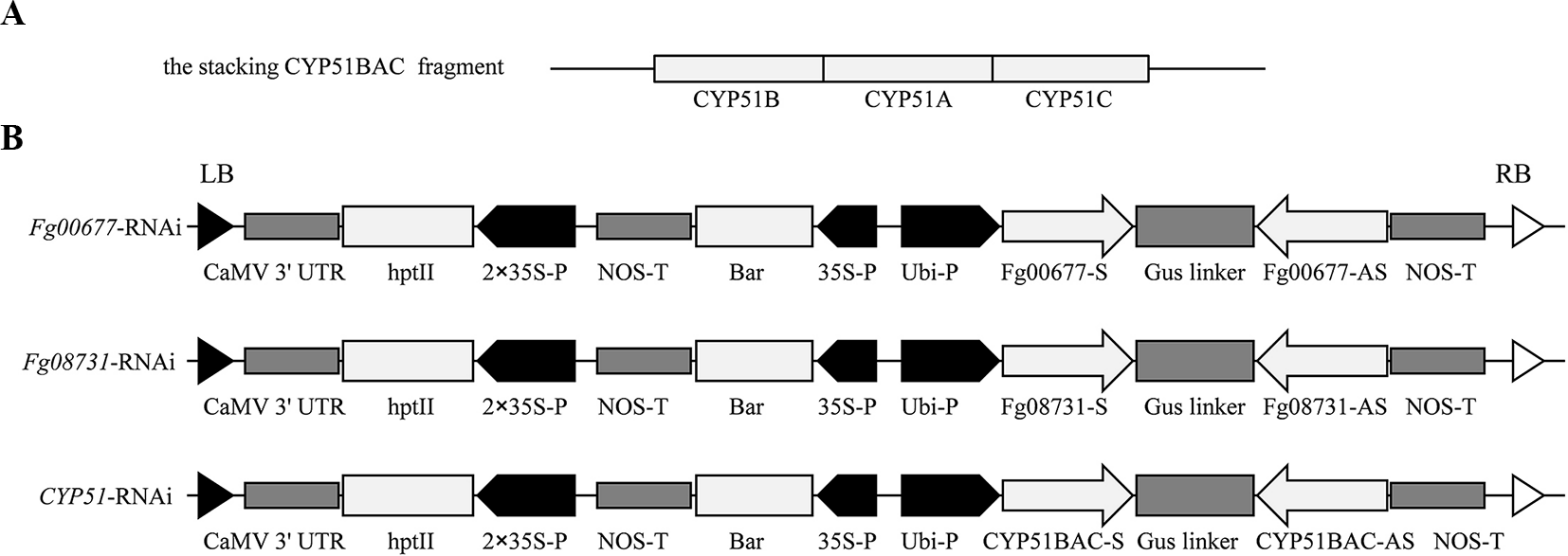
Structure of the RNAi construct. **(A)** The *CYP51BAC* fragment was generated by fragment stacking strategy from *CYP51A*, -*B*, and -*C* partial sequences for CYP51-RNAi cassette construction; **(B)** structure of the *Fg00677*-RNAi, *Fg08731*-RNAi, and *CYP51*-RNAi cassette for stable *B. distachyon* transformation. Each fragment derived from *Fg00677*, *Fg08731*, and *CYP51* in the sense (S) and antisense (AS) orientations was constructed such that the gus linker sequence was inserted between the S and AS sequences. Ubi-P, maize ubiquitin promoter; hpt II, hygromycin resistance gene; 2 × 35S-P, 2× CaMV 35S Promoter; Nos-T, Nos terminator; Bar, Biolaphos resistance gene.

**Figure 3:**
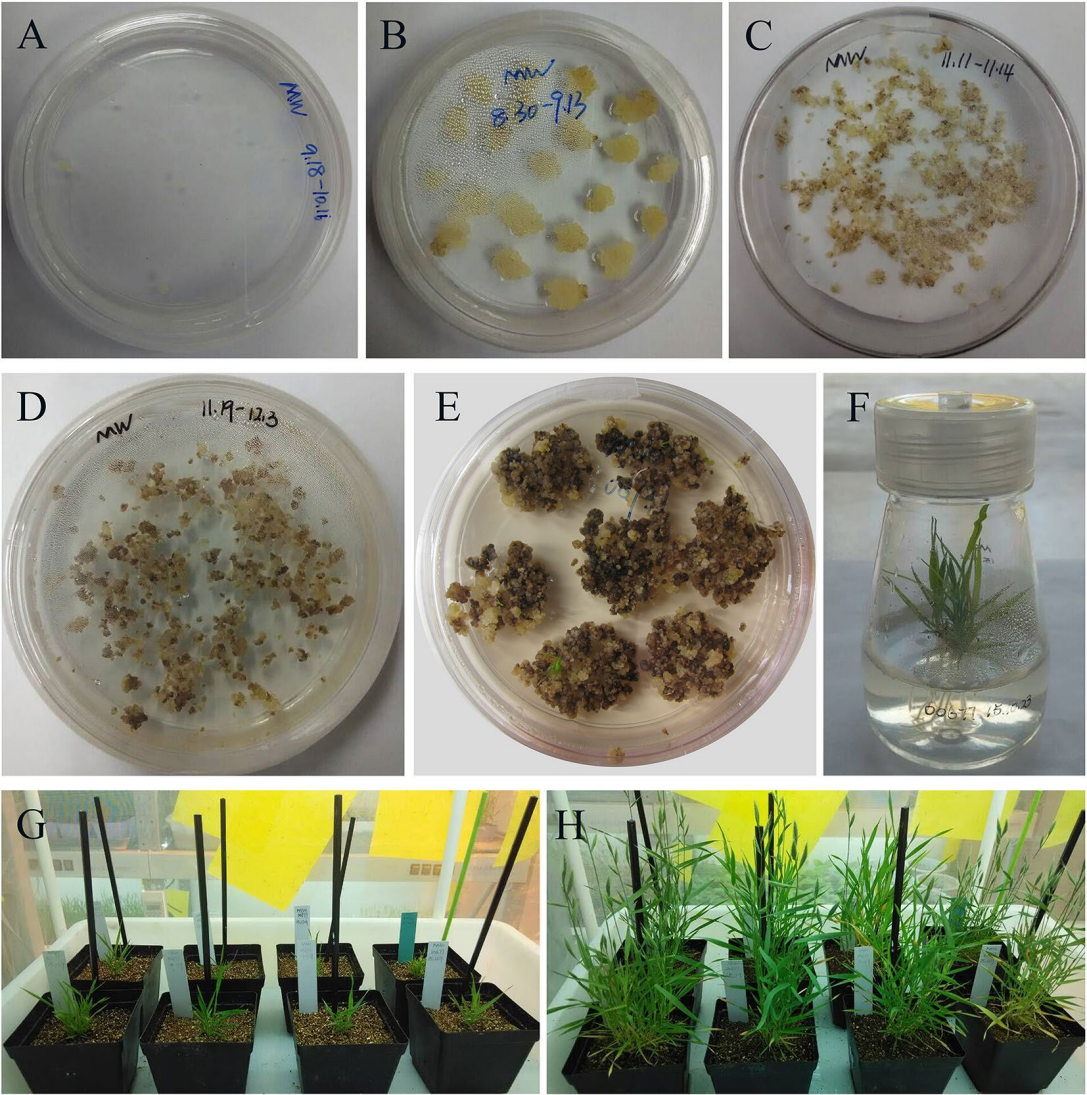
Stable genetic transformation of *B. distachyon* (genotype Bd21-3). **(A)** Immature embryos grown on callus induction medium; **(B)** embryo after 3 weeks on callus induction medium; **(C)** co-cultivation compact embryogenic callus with *Agrobacterium* on filter paper; **(D)** co-cultured compact embryogenic callus on selection medium; **(E)** regenerating callus on regeneration medium; **(F)** rooted putative transformants on rooting medium; putative transformant grown in soil for 2 weeks **(G)** and 8 weeks **(H)**, respectively.

**Figure 4:**
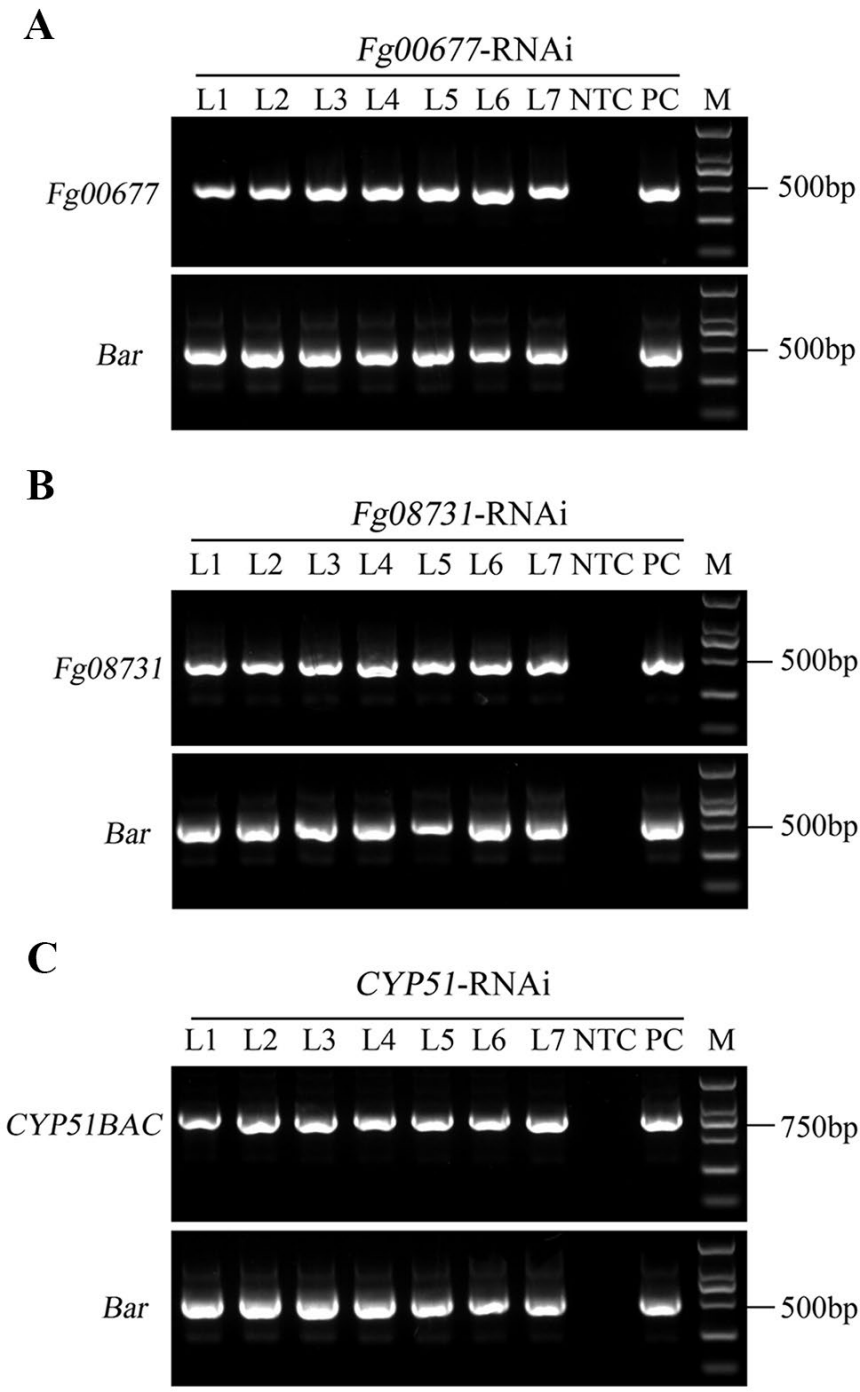
Molecular analysis of transgenic *B. distachyon*. **(A)** Integration of *Fg00677*-RNAi construct in transgenic plants analyzed by PCR amplification using *Fg00677* and *Bar* gene-specific primers. **(B)** Integration of *Fg08731*-RNAi construct in transgenic plants analyzed by PCR amplification using *Fg08731* and *Bar* gene-specific primers. **(C)** Integration of *CYP51*-RNAi construct in transgenic plants analyzed by PCR amplification using *CYP51BAC* and *Bar* gene-specific primers. The genomic DNA from non-transformed control (NTC) plants was used as negative control. The plasmid DNA, including FG00677-RNAi construct, FG08731-RNAi construct, or *CYP51*-RNAi construct was used as positive control (PC); M indicates DL2000 DNA marker.

Koch et al. (2013) confirmed that expression of CYP3RNA, a double-stranded (ds) RNA complementary to *CYP51A*, *CYP51B*, and *CYP51C*, caused co-silencing of *CYP51A*, *CYP51B*, and *CYP51C* in *F. graminearum*, thereby effectively inhibiting pathogen development and enhancing resistance in *Arabidopsis* and barley. The CYP3RNA was selected as the positive control to evaluate the efficiency of disease resistance in *B. distachyon*. The stacking *CYP51BAC* fragment, including 220 bp of *CYP51B*, 294 bp of *CYP51A*, and 238 bp of *CYP51C* was constructed in the pCAMBIA1300-based vector to produce *CYP51*-RNAi, which was transformed into *B. distachyon* Bd21-3 to express the same CYP3RNA as reported by Koch et al. (2013) (**Figure 2A**). To identify whether *Fg00677* and *Fg08731* are HIGS-effective targets, the selected 452 and 481 bp fragments from *Fg00677* and *Fg08731* were constructed into the pCAMBIA1300-based vector to produce *Fg00677*-RNAi and *Fg08731*-RNAi construct, respectively (**Figure 2B**; **Figure S2**). No off-targets were detected in *F. graminearum*, *B. distachyon*, wheat, and *Homo sapiens* for the selected fragments of *Fg00677* and *Fg08731* (**Table S2**). Each of these constructs contained an inverted repeat that, after transcription, is expected to result in a dsRNA sequence with a hairpin structure. Transgenic lines containing the RNAi construct were generated in the *B. distachyon* Bd21-3 by *Agrobacterium*-mediated transformation (**Figure 3**). In total, 21 putative T0 transgenic plants were produced from several transformation experiments. Molecular analysis of putative transgenic plants from T2 generations was performed by PCR analysis, which confirmed the presence of 452, 481, and 752 bp amplicons from the *Fg00677*, *Fg08731*, and *CYP51BAC* in the corresponding T2 transgenic line. No amplification bands were observed in the non-transgenic control plants (**Figure 4**). In addition, integration of the *Bar* gene segment in T2 transgenic plants was verified by PCR (**Figure 4**). The result indicated that the *Fg00677*-RNAi, *Fg08731*-RNAi, and *CYP51*-RNAi constructs were integrated into the *B. distachyon* plant genome successfully by *Agrobacterium-*mediated transformation.

### Production of siRNAs in Transgenic *B. distachyon* Lines

**Figure 5:**
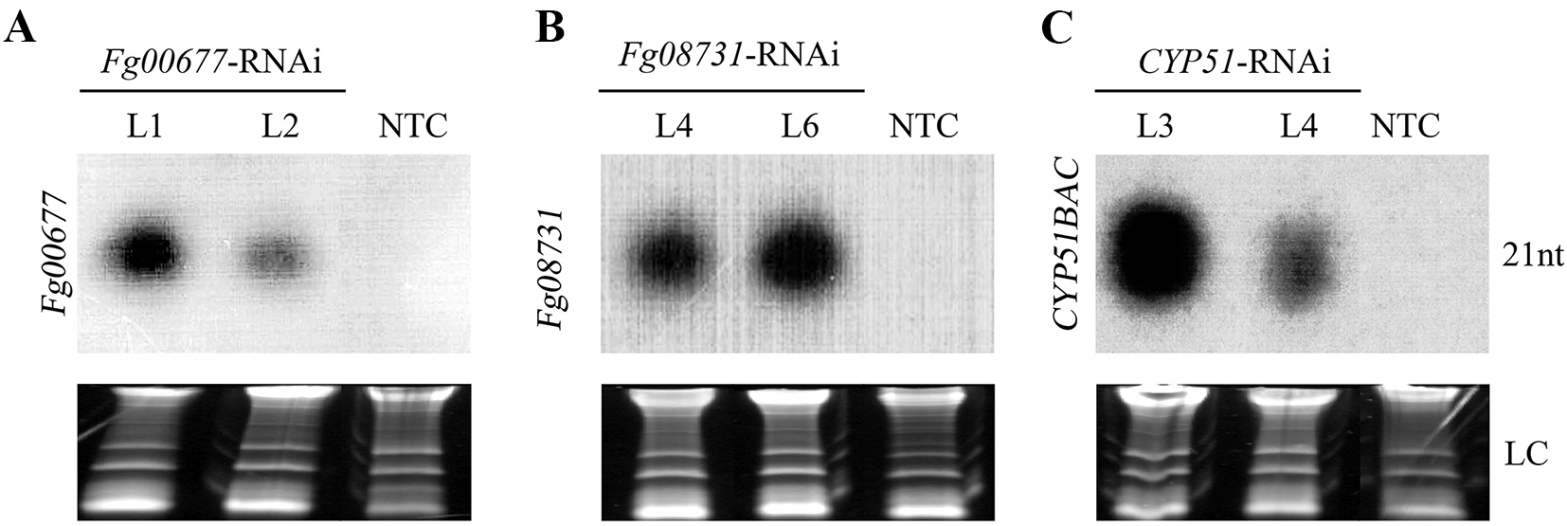
siRNA detection in transgenic *B. distachyon* lines. Northern blot analysis of sequence-specific siRNA molecules derived from *Fg00677* **(A)**, *Fg08731* **(B)**, and *CYP51BAC* **(C)** RNAi fragments in the T2 transgenic *B. distachyon* lines with *Fg00677*-RNAi, *Fg08731*-RNAi, and *CYP51*-RNAi constructs. Ethidium bromide-stained rRNA served as loading control (LC). NTC, non-transformed control.

To determine the presence of homologous siRNA in transgenic *Fg00677*-RNAi, *Fg08731*-RNAi, and *CYP51*-RNAi lines, northern blot analysis was performed using the sequence-specific probes. The result showed that the siRNA molecules derived from *Fg00677*-RNAi, *Fg08731*-RNAi, and *CYP51BAC*-RNAi constructs were present in the transgenic T2 lines (*Fg00677*-RNAi-L1, -L2, *Fg08731*-RNAi -L3, -L4, -L6, *CYP51*-RNAi -L3, -L4) prior to *F. graminearum* inoculation (**Figure 5**; **Figure S3**). No hybridization signal was detected in the other transgenic T2 lines (*Fg00677*-RNAi -L3, -L4, -L5, -L6, -L7, *Fg08731*-RNAi –L1, -L2, -L5, -L7 and *CYP51*-RNAi -L1, -L2, -L5, -L6, -L7) or non-transformed control plants (**Figure S3**). The results showed that some RNAi transgenic plants express enough siRNA, and the other RNAi transgenic plants could not express enough siRNAs which can be detected by northern blot. In addition, the results indicated that the dsRNA derived from respective *Fg00677*-RNAi, *Fg08731*-RNAi, or *CYP51*-RNAi constructs were produced in the transgenic plants and processed by the host silencing machinery into siRNA molecules. Thus, the transgenic *B. distachyon* lines (*Fg00677*-RNAi-L1, -L2, *Fg08731*-RNAi -L4, -L6, *CYP51*-RNAi -L3, -L4) which produced more siRNAs were selected for further analysis.

### Response of Transgenic *B. distachyon* Plants Inoculated With *F. graminearum*

**Figure 6:**
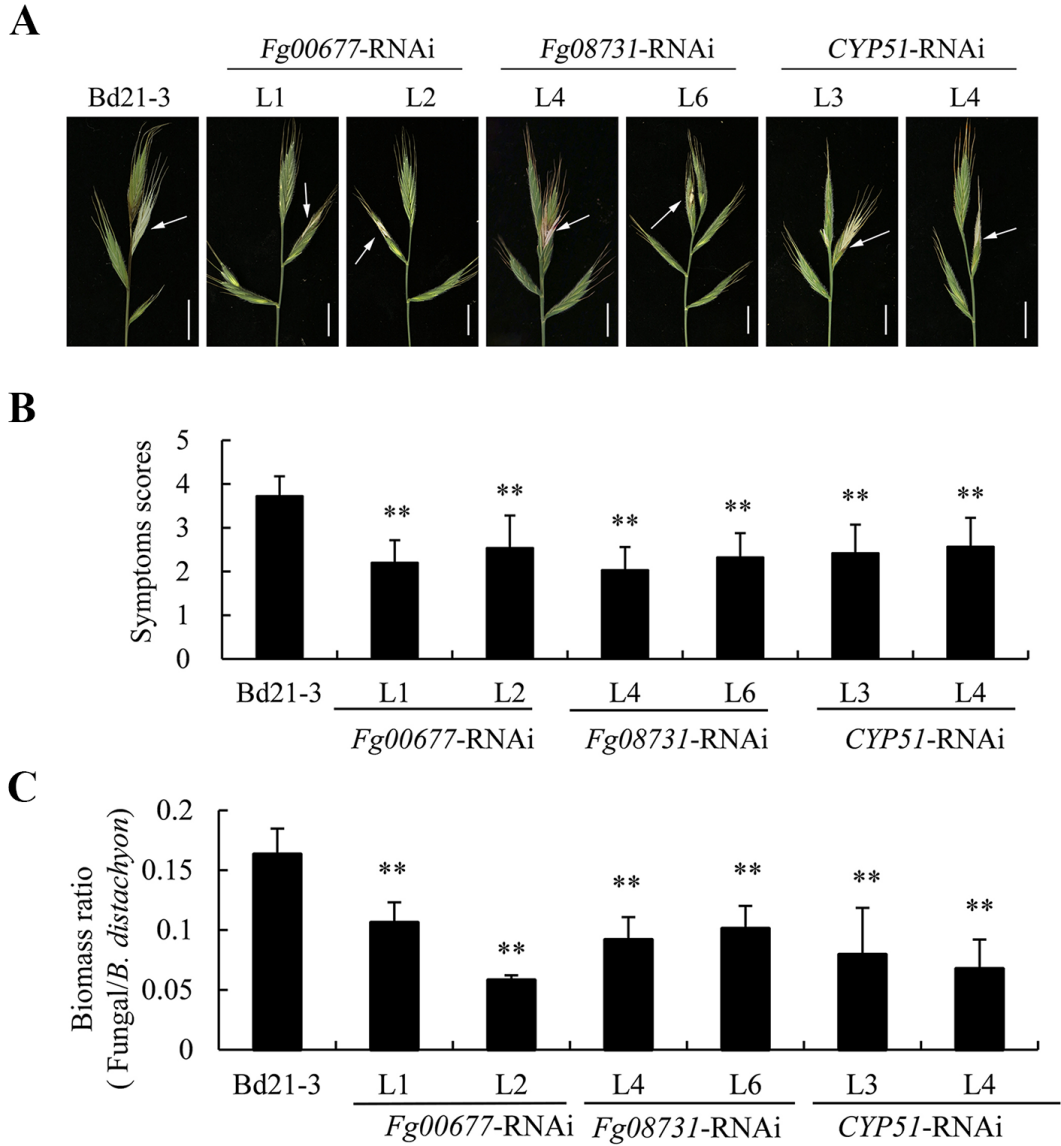
Evaluation of disease resistance of T2 transgenic *B. distachyon* lines with *Fg00677*-RNAi, *Fg08731*-RNAi, and *CYP51*-RNAi constructs against *F. graminearum*. **(A)** The second spikelet from the top of the spike of transgenic *B. distachyon* lines and non-transgenic controls was point inoculated with *F. graminearum* PH-1 strain and photographed at 9 dpi. Bar, 1 cm. Arrows indicate the inoculation site. **(B)** Quantification of the disease symptoms using a scoring scale at 9 dpi. Values represent the means ± standard deviation of at least 30 plants. Similar results were obtained from three biological replicates. **(C)** *F. graminearum* and *B. distachyon* biomass ratio measured *via* total DNA content at 5 dpi by absolute quantification using the internal reference genes *beta-tubulin* and *BdUBC18*, respectively. Values represent the means ± standard deviation of three biological replicates. Differences were assessed using Student’s *t* tests. Double asterisks indicate *P* < 0.01.

The transgenic *B. distachyon* plants that contained RNAi constructs displayed normal morphology (**Figure S4**), indicating no unintended effects in the RNAi plants. Two independent transgenic lines of the T2 generations were assayed for their resistance to *F. graminearum* strain PH-1 wild type of Bd21-3 was also inoculated and served as the control. At the flowering stage, the spikes of T2 transgenic plants of the same lines were inoculated by single-floret injection. After inoculations, obvious differences in disease symptoms on spikes were clearly discernible between the transgenic plants and the non-transgenic plants (**Figure 6A**). In the transgenic plants with *Fg00677*-RNAi, *Fg08731*-RNAi, and *CYP51*-RNAi constructs, the FHB symptom occurred only in the partial florets of the inoculated spikelet. However, symptoms appeared on the entire inoculated spikelet and at least one adjacent spikelet in the non-transformed line Bd21-3 (**Figure 6A**). To evaluate the extent of spike colonization, the scoring method described by Pasquet et al. (2016) was used for scoring the symptoms at 9 days post-inoculation (dpi). The transgenic *B. distachyon* lines (*Fg00677*-RNAi -L1, -L2, *Fg08731*-RNAi -L4, -L6, *CYP51*-RNAi -L3, -L4) showed average disease symptom grades of 2.20, 2.54, 2.03, 2.32, 2.42, and 2.57, respectively, significantly lower than that of Bd21-3 which exhibited an average disease symptom grade of 3.72 (**Figure 6B**). The biomass of *F. graminearum* showed a significant decrease in lines *Fg00677*-RNAi -L1, -L2, *Fg08731*-RNAi -L4, -L6, *CYP51*-RNAi -L3, -L4 compared with Bd21-3 (**Figure 6C**; **Figure S5**). These results indicated significant increased resistance to *F. graminearum* in transgenic lines compared with that in the control plants.

### Reduced Transcript Levels of the Target Genes in the Transgenic Plants Inoculated With *F. graminearum*

**Figure 7:**
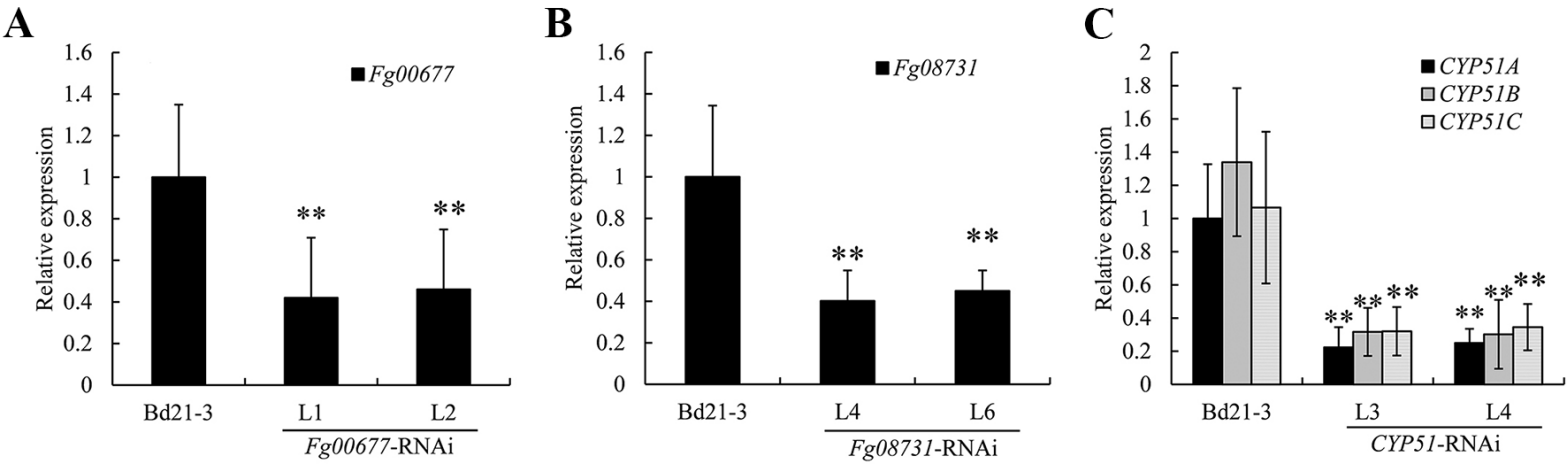
Relative transcript levels of *Fg00677* **(A)**, *Fg08731* **(B)**, *CYP51A*, *CYP51B*, and *CYP51C* **(C)** in *F. graminearum*-inoculated spikelets. The expression values were normalized to the level of the *beta-tubulin* of *F. graminearum*, and the expression level of target genes in *F. graminearum*-inoculated non-transformed plants set at 1. Bars represent the means ± standard deviation of three biological replicates. Differences were assessed using Student’s *t* test. Double asterisks indicate *P* < 0.01.

To determine the effect of HIGS strategy on the targeted *F. graminearum* genes (*Fg00677*, *Fg08731*, *CYP51A*, *CYP51B*, and *CYP51C*), we measured the relative transcript levels of these genes in the pathogens infecting transgenic lines and compared them with the levels in pathogens infecting non-transformed plants. Total RNA was isolated from infected plant tissue 72 h after inoculation with *F. graminearum* PH-1 and cDNA was synthesized by reverse transcription. Gene-specific primers were designed for *Fg00677*, *Fg08731*, *CYP51A*, *CYP51B*, and *CYP51C* outside the fragments used in the RNAi constructs to avoid possible artifacts. The transcript levels of *F. graminearum beta-tubulin* were also measured and used for normalization. Analysis of two independent transgenic lines showed a clear down-regulation of *Fg00677*, *Fg08731*, *CYP51A*, *CYP51B*, and *CYP51C* in *F. graminearum* infecting transgenic *B. distachyon* lines as compared to *F. graminearum* infecting non-transgenic controls (**Figure 7**). In the *F. graminearum*-infected seedlings, the *Fg00677* transcripts were reduced by 58% and 54%, respectively, in the two *Fg00677*-RNAi transgenic lines L1 and L2 relative to control seedlings (**Figure 7A**). Similarly, the *Fg08731* transcripts were reduced by 60% and 55%, respectively, in the two *F. graminearum*-infected *Fg08731*-RNAi transgenic lines L4 and L6 relative to control seedlings (**Figure 7B**). The *CYP51A*, *CYP51B*, and *CYP51C* transcripts were reduced by 78%, 77%, and 70% in the *F. graminearum*-infected *CYP51*-RNAi transgenic line L3, and reduced by 75%, 78%, and 67% in the *F. graminearum*-infected transgenic line L4, respectively, relative to *F. graminearum*-infected control seedlings (**Figure 7C**). The results showed that the transcript levels of *Fg00677*, *Fg08731*, *CYP51A*, *CYP51B*, and *CYP51C* were reduced significantly in fungi infecting corresponding transgenic lines. The results further indicated that the significant increased resistance to *F. graminearum* was due to the reduced transcript levels of corresponding genes in the corresponding transgenic plants with RNAi cassette.

## Discussion

Genetic breeding for FHB disease resistance represents the most cost-effective control strategy. However, resistance breeding for FHB and reduced deoxynivalenol (DON) accumulation is complex and difficult, due to the limited disease-resistant germplasm sources and the rapid breakdown of resistance by new virulence races (Wegulo et al., 2015; Machado et al., 2018). Recently, HIGS has been applied in more plant pathogens, including FHB fungus *F. graminearum*, and provided a new strategy for the safe, pesticide-free and potentially sustainable plant protection against plant pathogen (Koch et al., 2013; Cheng et al., 2015; Chen et al., 2016). The objective of this study was to identify new effective HIGS target genes, and determine whether *B. distachyon* could be used as a model plant to identify the effective HIGS target of *F. graminearum* for using HIGS technology.

Increasing resistance against pathogens through HIGS technology in host depends on the effective HIGS targets which play a vital role in growth, development, and pathogenicity in fungi. Protein kinases play an important regulatory role in the pathogenesis and growth of plant pathogens. Earlier, it was reported that the two protein kinases encoding genes, *CK2* orthologous gene *Fg00677* and the *CK1* orthologous gene *Fg08731*, are essential in *F. graminearum*, because deletion of them is lethal (Wang et al., 2011). *CK2* and *CK1* orthologous genes also play important roles in other organisms, especially in fungi. CK2 is involved in numerous cellular processes, including cellular morphology, signal transduction, ion homeostasis, cell viability, cellular polarity, and cell cycle progression (Tripodi et al., 2007; Lei et al., 2014; Wang et al., 2015). CK2 is essential for regulating cell cycle progression and proliferation in *S. cerevisiae* and *S. pombe* (Snell and Nurse, 1994; Rethinaswamy et al., 1998), and is required for growth and development in two filamentous fungi: *N. crassa* and *A. nidulans* with mutants resulting in microcolony and aerial mycelium-defective phenotype (Yang et al., 2002; He et al., 2006; Mehra et al., 2009; de Souza et al., 2013). CK1 enzymes have important roles in regulating several cellular processes, such as cell cycle, cell division, and cell differentiation, and the circadian clock in mammals, *Drosophila*, and *S. cerevisiae* (Brockman et al., 1992; Eide et al., 2005; Knippschild et al., 2005). CK1 orthologs characterized in *S. cerevisiae*, *A. nidulans*, and *Neurospora crassa* are proven to be essential for growth and development (Hoekstra et al., 1991; Hoekstra et al., 1994; Zhou and Elledge, 2000; Park et al., 2011; Apostolaki et al., 2012). Therefore, we selected these two genes for further analysis. We obtained the full-length ORFs of *Fg00677* and *Fg08731* from *F. graminearum* by PCR. Sequence analysis indicated that *Fg00677* and *Fg08731* contain the STKc_CK2_alpha conserved domain and STKc_CK1_delta_epsilon conserved domain, respectively. Multiple-alignment and phylogenetic analysis indicated that *Fg00677* and *Fg08731* are highly conserved in filamentous fungi. It is well known that the evolutionarily conserved proteins will play the critical functions usually. Therefore, *Fg00677* and *Fg08731* may be the effective HIGS target, which were identified using HIGS technology subsequently.

RNAi refers to a biological phenomenon that is capable of causing sequence-specific inhibition of gene expression at the transcriptional, post-transcriptional, or translational levels (Baulcombe, 2015). RNAi exists in plants, and the RNAi mechanism is conserved and widely involved in the growth and development of organisms (Plasterk, 2002). RNAi also exists in fungi, and the Dicer-dependent RNAi machinery was discovered in *F. graminearum* (Chen et al., 2015; Son et al., 2017). HIGS relies on the plant’s RNAi system to produce silencing molecules (dsRNA and siRNAs) which can be translocated into fungus through unclear mechanism, then relies on the interacting RNAi system of the fungus to down-regulate the expression of fungal genes (Cai et al., 2018). In this study, the siRNAs complementary to the respective fungal gene were detected by northern blot in the transgenic *B. distachyon* carrying the respective RNAi construct, indicating that dsRNAs were produced through transcription in the transgenic *B. distachyon*, and the dsRNAs were processed into siRNAs by plant’s RNAi system. The endogenous transcripts of the HIGS target genes (*Fg00677*, *Fg08731*, *CYP51A*, *CYP51B*, and *CYP51C*) were reduced in *F. graminearum* colonizing transgenic *B. distachyon* spikes carrying the respective RNAi construct, suggesting that the dsRNAs or siRNAs produced by transgenic *B. distachyon* may be translocated into the fungal cells during the infection process and serve as the silencing molecules that initiated *in vivo* post-transcriptional gene silencing in the colonizing fungus.

The reduction of the respective gene’s transcripts in colonizing *F. graminearum* by transgenic *B. distachyon* T2 lines expressing HIGS cassettes correlates with significant protection of *B. distachyon* against the disease. The resistance of *Fg00677*-RNAi and *Fg08731*-RNAi T2 lines reached the level equivalent to *CYP51*-RNAi lines. The engineered resistance trait existed in the T2 generation, indicating that the disease resistance traits can be transmitted to next generations. The HIGS cassettes of *Fg00677* and *Fg08731* do not contain an effective target of host genes in the *B. distachyon* genomic sequence according to the off-target prediction tool; thus, the probability of off-target silencing of the host gene is low. In this study, we did not observe a difference in growth status between the transgenic *B. distachyon* plants and the wild type Bd21-3. Although the expression levels of *Fg08731* and *Fg00677* were reduced partially in colonizing *F. graminearum*, the FHB resistance was enhanced significantly, indicating that the degree of silencing by HIGS is sufficient to attenuate pathogenicity of *F. graminearum*. Although *Fg00677* and *Fg08731* are essential in *F. graminearum*, we did not observe complete resistance to *F. graminearum* as the smaller browning or bleaching lesion occurred on the inoculated spikelet. This may be due to some degree of pathogenicity conferred by the residual transcripts, indicating that exploring HIGS technology for effectively improving disease resistance requires sufficient silencing of the target genes of phytopathogens. To increase the degree of resistance to pathogens, multiple genes can be silenced. Koch et al. (2013) and our study found that simultaneously silencing all three *CYP51* genes in *F. graminearum* by HIGS completely eliminates fungal pathogenicity. Therefore, simultaneously silencing *Fg00677* and *Fg08731* may confer the enhanced resistance to *F. graminearum*. In conclusion, *Fg00677* and *Fg08731* are effective HIGS targets which can be used for improving resistance against FHB disease in wheat and other cereal crops.

The key genes of plant pathogen identified by knock-out technology were considered as candidate HIGS targets. However, whether these genes can be silenced effectively using HIGS technology is uncertain as the current mechanism of HIGS is not yet clear. Therefore, the candidate HIGS target genes need be validated in a plant-pathogen interaction system before being applied in disease-resistant breeding in wheat. However, it is not feasible to use transgenic wheat in screening of effective HIGS targets, because wheat genetic transformation is time consuming and costly, and wheat has a long growth cycle. *B. distachyon*-*F. graminearum* interactions closely model the head blight in wheat and barley caused by *F. graminearum* (Peraldi et al., 2011). *B. distachyon* as a grass crop, may be used as alternative plant for batch screening of HIGS targets, due to the shorter growth cycle and easier genetic transformation compared with wheat and other cereal crops. Koch et al. (2013) showed that HIGS targeting of the CYP51 genes rendered susceptible plants highly resistant to *F. graminearum* infection in *Arabidopsis* and barley. In this study, HIGS of CYP51 genes also confers efficient resistance against *F. graminearum* infection in *B. distachyon*. In addition, HIGS of two essential protein kinase encoding genes (*Fg00677* and *Fg08731*) enhanced the resistance against *F. graminearum* infection in *B. distachyon*, significantly. Our data indicated that HIGS can be used in *B. distachyon*-​*F. graminearum* interactions, and *B. distachyon* can be used as a model plant for identifying the effective HIGS targets of *F. graminearum*, with providing time and money saving benefits compared with wheat.

## Data Availability Statement

All datasets for this study are included in the article/**Supplementary Material**.

## Author Contributions

JG and ZK designed the experiment. FH, RZ, and JZ performed the experiments and analyzed the data. FH, TQ, and JG wrote the manuscript.

## Funding

This study was supported by the National Key R&D Program of China (2018YFD0200400), Natural Science Basic Research Plan in Shaanxi Province of China (2017JM3007), and National Natural Science Foundation of China (31620103913).

## Conflict of Interest

The authors declare that the research was conducted in the absence of any commercial or financial relationships that could be construed as a potential conflict of interest.

## Supplementary Material

Figure S1 Amino acid sequence alignments of Fg00677 (A) and Fg08731 (B) with other fungal homologs. Amino acid identity (black boxes) and similarity (gray boxes) are shown within the protein kinase domain. The active sites are highlighted with a filled triangle. The ATP binding site is indicated with an arrow. Asterisks indicate the activation loop. Thick black horizontal line indicates the conserved domain of the corresponding protein kinase.

Figure S2 The nucleotide sequences of Fg00677 and Fg08731 used for RNAi constructs. The full-length sequences represent the ORFs of Fg00677 (A) and Fg08731 (B). The nucleotides highlighted with bulue color of Fg00677 (A) and Fg08731 (B) were used for RNAi constructs.

Figure S3 siRNA detection in transgenic B. distachyon lines. Northern blot analysis of sequence-specific siRNA molecules derived from Fg00677, Fg08731, and CYP51BAC RNAi fragments in the T2 transgenic B. distachyon lines with Fg00677-RNAi, Fg08731-RNAi, CYP51-RNAi constructs. Ethidium bromide-stained rRNA served as loading controls (LC). NTC, non-transformed control.

Figure S4 Phenotypes of the Fg00677-RNAi, Fg08731-RNAi, and CYP51-RNAi lines. Transgenic lines compared with the wild-type Bd21-3 at 28 days (A) and at 40 days after sowing (B). Bars, 5 cm.

Figure S5 The standard curve generated for the absolute quantification of F. graminearum and B. distachyon. The fragments of beta-tubulin and BdUBC18 were fused to the pMD18-T vector to generate the templates. Threshold cycles were plotted against the quantity of the templates. (A) Standard curve for beta-tubulin generated by using the quantity of the templates (101, 102, 103, 104, 105, 106, 107, 108). (B) Standard curve for BdUBC18 generated by using the quantity of the templates (104, 105, 106, 107, 108).

Table S1 List of primers used in this study.

Table S2 Prediction of off-target transcripts for Fg00677 and Fg08731.
