# Supplementary figures and images for: Host-Induced Silencing of Fusarium graminearum Genes Enhances the Resistance of Brachypodium distachyon to Fusarium Head Blight
Source: Front Plant Sci. 2019 Oct 30;10:1362. doi: 10.3389/fpls.2019.01362 (PMC6831556; doi:10.3389/fpls.2019.01362)

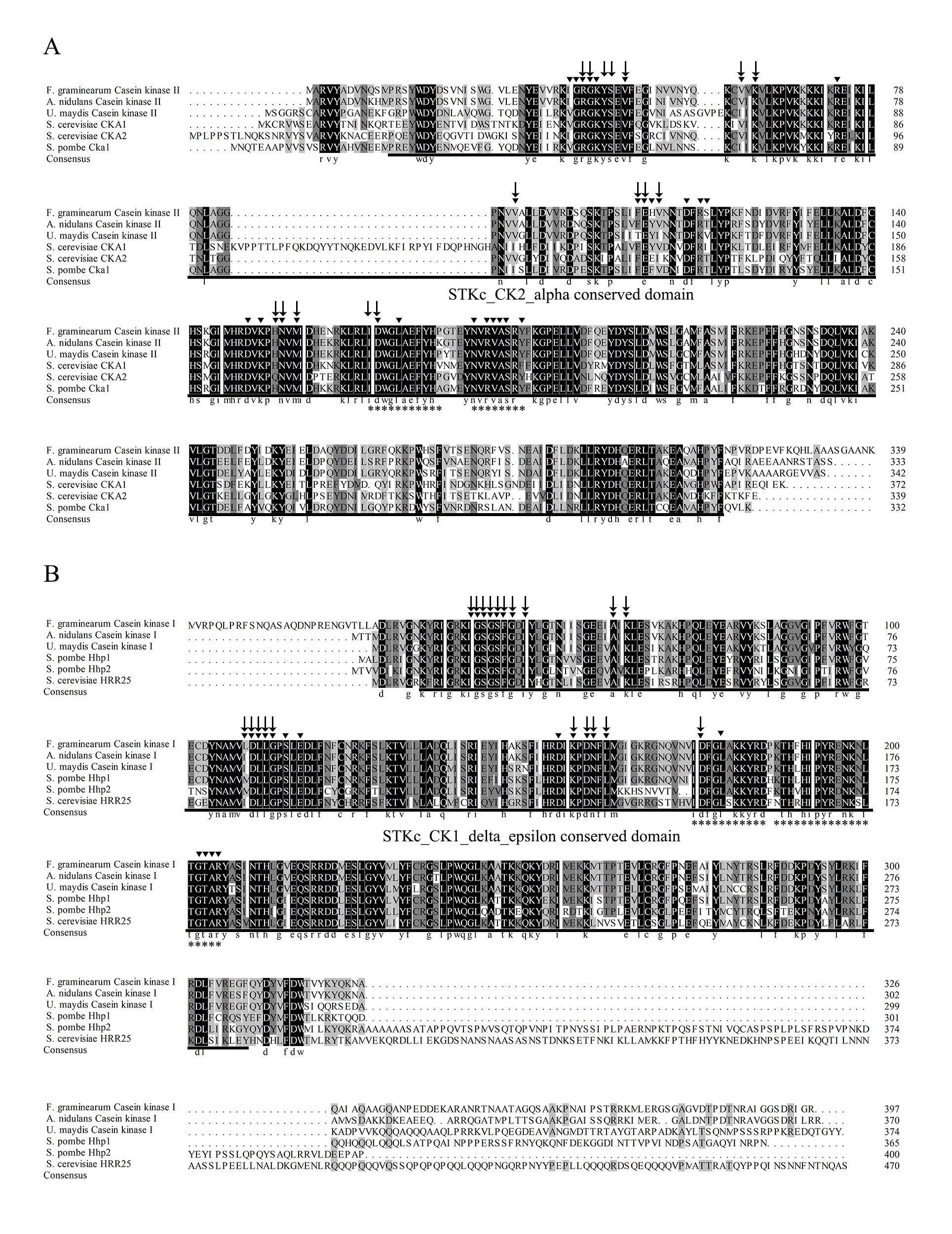

Supplement: Figure S1 — Amino acid sequence alignments of Fg00677 (A) and Fg08731 (B) with other fungal homologs. Amino acid identity (black boxes) and similarity (gray boxes) are shown within the protein kinase domain. The active sites are highlighted with a filled triangle. The ATP binding site is indicated with an arrow. Asterisks indicate the activation loop. Thick black horizontal line indicates the conserved domain of the corresponding protein kinase. [file Image_1.jpeg]

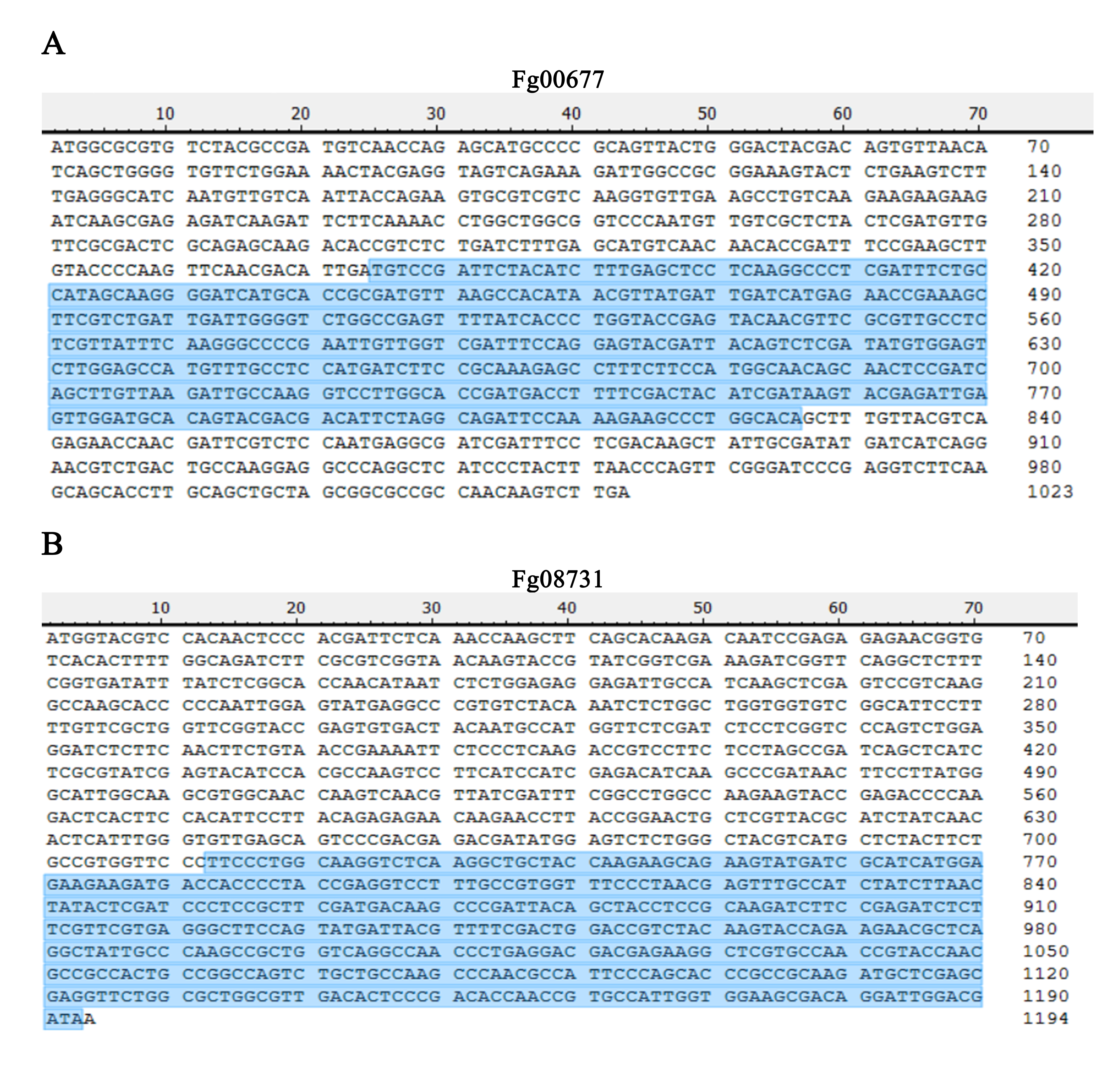

Supplement: Figure S2 — The nucleotide sequences of Fg00677 and Fg08731 used for RNAi constructs. The full-length sequences represent the ORFs of Fg00677 (A) and Fg08731 (B). The nucleotides highlighted with bulue color of Fg00677 (A) and Fg08731 (B) were used for RNAi constructs. [file Image_2.jpeg]

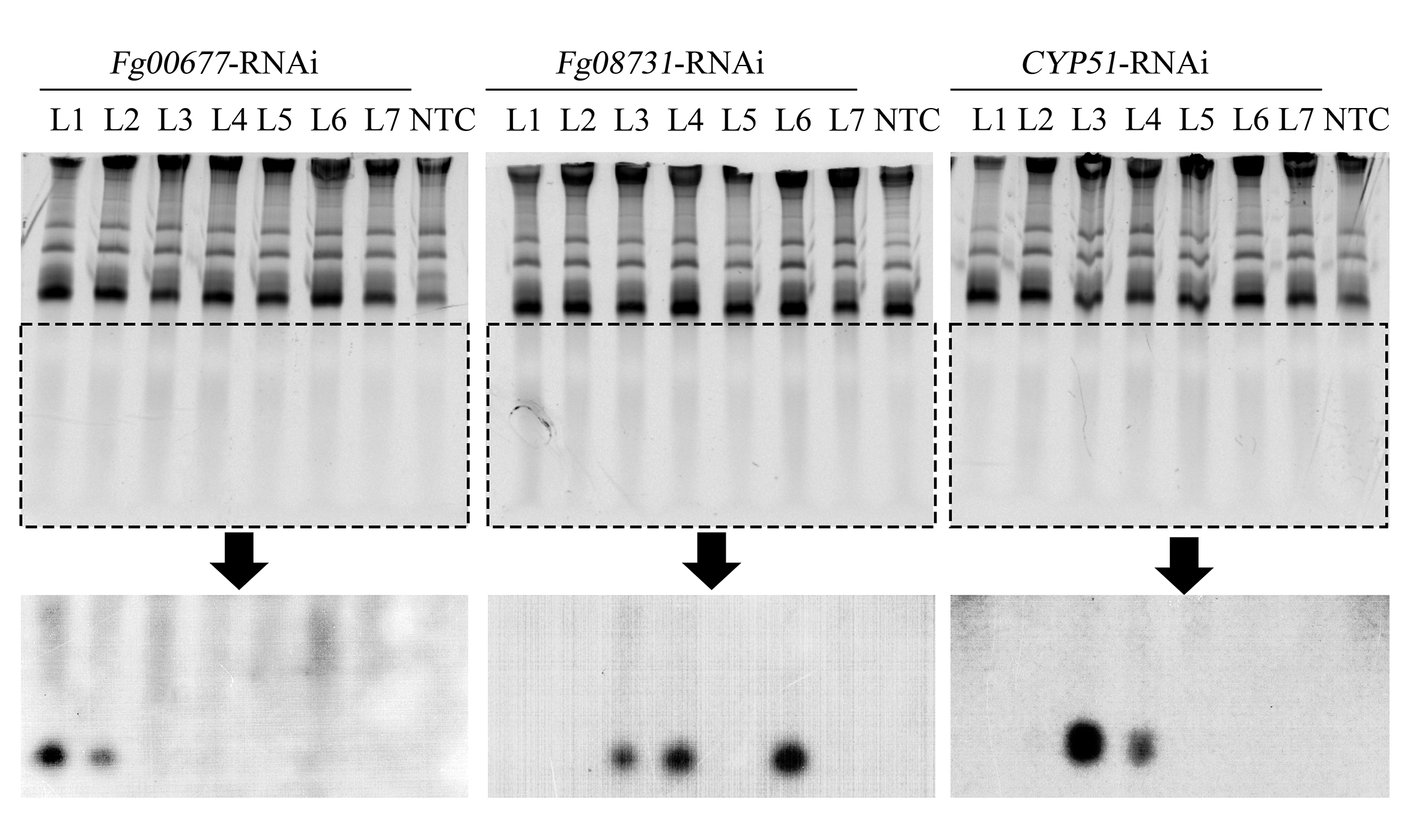

Supplement: Figure S3 — siRNA detection in transgenic B. distachyon lines. Northern blot analysis of sequence-specific siRNA molecules derived from Fg00677, Fg08731, and CYP51BAC RNAi fragments in the T2 transgenic B. distachyon lines with Fg00677-RNAi, Fg08731-RNAi, CYP51-RNAi constructs. Ethidium bromide-stained rRNA served as loading controls (LC). NTC, non-transformed control. [file Image_3.jpeg]

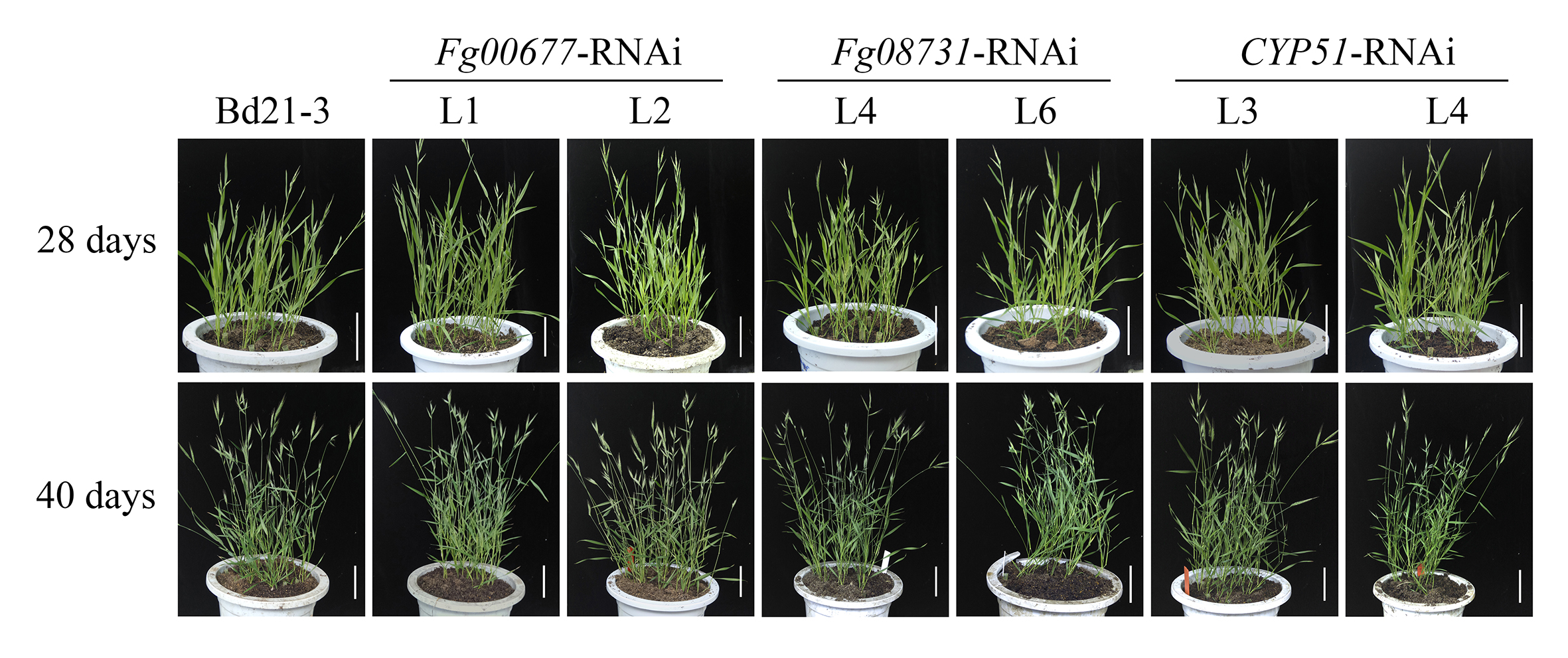

Supplement: Figure S4 — Phenotypes of the Fg00677-RNAi, Fg08731-RNAi, and CYP51-RNAi lines. Transgenic lines compared with the wild-type Bd21-3 at 28 days (A) and at 40 days after sowing (B). Bars, 5 cm. [file Image_4.jpeg]

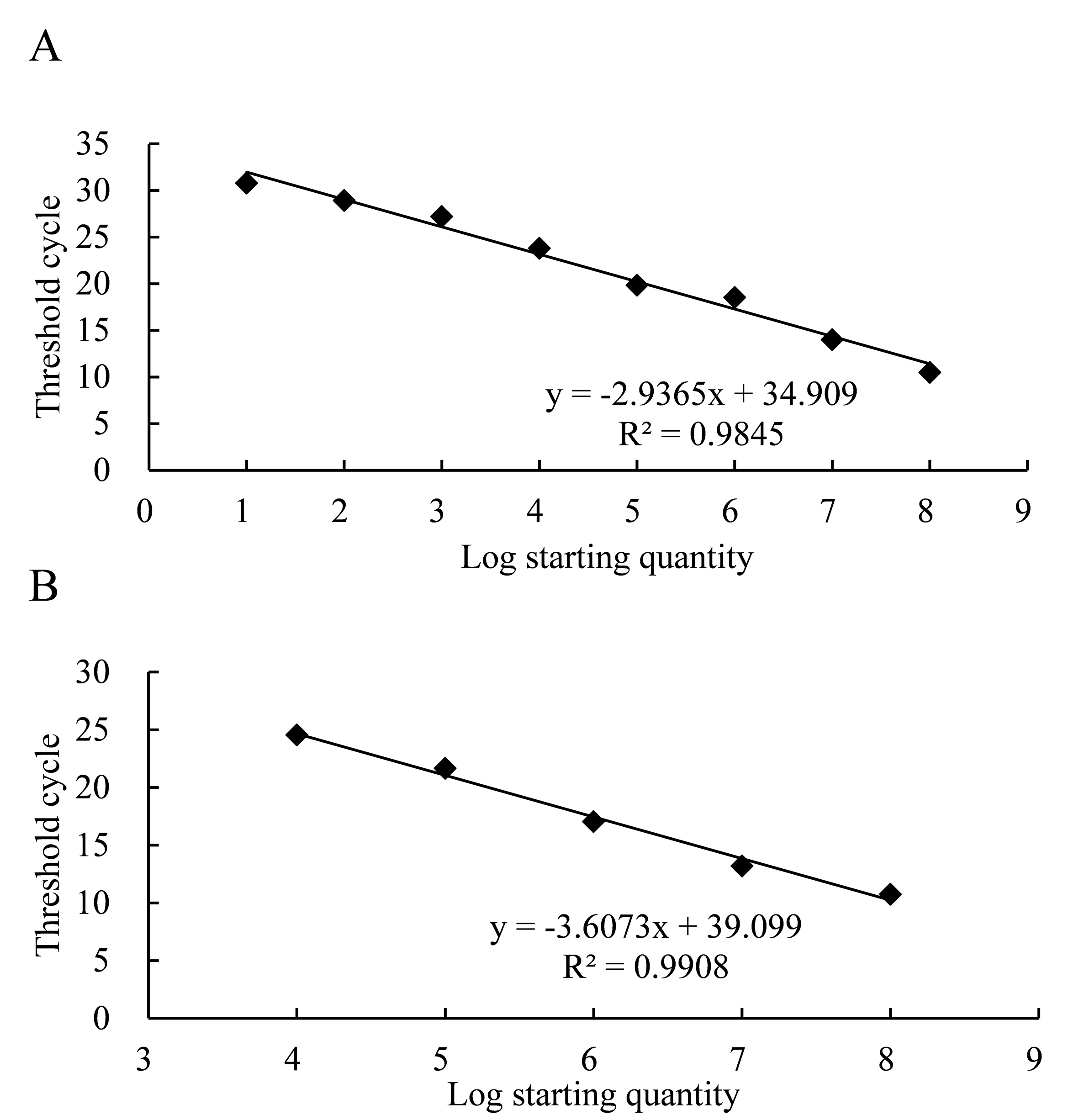

Supplement: Figure S5 — The standard curve generated for the absolute quantification of F. graminearum and B. distachyon. The fragments of beta-tubulin and BdUBC18 were fused to the pMD18-T vector to generate the templates. Threshold cycles were plotted against the quantity of the templates. (A) Standard curve for beta-tubulin generated by using the quantity of the templates (101, 102, 103, 104, 105, 106, 107, 108). (B) Standard curve for BdUBC18 generated by using the quantity of the templates (104, 105, 106, 107, 108). [file Image_5.jpeg]
